# Long-term recovery and microbiota–gut–brain axis disruption after traumatic brain injury (OVERCOME-TBI): protocol for a prospective, observational clinical study

**DOI:** 10.1136/bmjopen-2026-120552

**Published:** 2026-09-23

**Authors:** Matti Artosalo, Anna Östberg, Olli Tenovuo, Timo Roine, Mehrbod Mohammadian, Teemu Kallonen, Kimmo Salminen, Teppo Stenholm, Antti Hakanen, Teija Sainio, Jussi Hirvonen, Alex M Dickens, Matej Orešić, András Büki, Tuulia Hyötyläinen, David Menon, Virginia Newcombe, Anne-Cécile Chiollaz, Jean-Charles Sanchez, Stefania Mondello, Laura Airas, Jussi P Posti

**Affiliations:** 1 Abdominal Centre – Gastroenterology, University of Turku and Turku University Hospital, Turku, Finland; 2 Neurocenter, Department of Neurosurgery and Turku Brain Injury Center, Turku University Hospital, Turku, Finland; 3 Clinical Neurosciences, University of Turku, Turku, Finland; 4 Department of Neuroscience and Biomedical Engineering, Aalto University School of Science, Espoo, Finland; 5 Department of Radiology, Athinoula A. Martinos Center for Biomedical Imaging, Massachusetts General Hospital, Harvard Medical School, Boston, Massachusetts, USA; 6 Department of Clinical Microbiology, Turku Clinical Microbiome Bank, Turku University Hospital, Turku, Finland; 7 Institute of Biomedicine, University of Turku, Turku, Finland; 8 Microbe Centre, University of Turku and Wellbeing Services County of Southwest Finland, Turku, Finland; 9 Department of Medical Physics, University of Turku and Turku University Hospital, Turku, Finland; 10 Department of Radiology, Turku University and Turku University Hospital, Turku, Finland; 11 Turku Bioscience Centre, University of Turku and Åbo Akademi University, Turku, Finland; 12 Department of Chemistry, University of Turku, Turku, Finland; 13 School of Medical Sciences, Faculty of Medicine and Health, Örebro University, Örebro, Sweden; 14 Department of Life Technologies, University of Turku, Turku, Finland; 15 School of Science and Technology, Örebro University, Örebro, Sweden; 16 Department of Medicine, University of Cambridge, Cambridge, UK; 17 Faculty of Medicine, University of Geneva, Geneva, Switzerland; 18 Department of Biomedical and Dental Sciences and Morphofunctional Imaging, University of Messina, Messina, Italy; 19 Turku PET Centre, University of Turku and Turku University Hospital, Turku, Finland

**Keywords:** Brain Injuries, Microbiota, Gastrointestinal Microbiome, NEUROSURGERY, NEUROLOGY

## Abstract

**Introduction:**

Traumatic brain injury (TBI) is a major cause of long-term disability worldwide, but the pathophysiological mechanisms underlying its chronic sequelae remain incompletely understood. In addition to persistent neuroinflammation, evidence suggests that dysregulation of the gut microbiota influences brain recovery. The OVERCOME-TBI study investigates the long-term functional, cognitive, imaging, biochemical and gastrointestinal outcomes after TBI of all severities, with the aim of identifying biomarkers and microbiome signatures associated with progressive neurodegeneration and poor recovery.

**Methods and analysis:**

This is a prospective, observational study with long-term follow-up of two previously established TBI cohorts—the Evidence-based Diagnostic and Treatment Planning Solution for Traumatic Brain Injuries (TBIcare; recruited 2011–2013) and the Pathophysiological Characterisation of Severe TBI (PACoS-TBI; recruited 2020–2023). The design combines prospectively collected acute and subacute data from the original cohorts with new cross-sectional assessments performed at chronic time points (2–5 and 12–15 years post-injury). Participants are re-contacted and re-consented for these chronic-phase assessments. Up to 160 patients with TBI and 30 orthopaedic injury controls will undergo multimodal examinations: neurological and neuropsychological testing, advanced MRI including multishell diffusion and resting-state functional MRI, positron emission tomography (PET) with the radioligand [^11^C]PK11195, blood-based proteomics, lipidomics, metabolomics, faecal and intestinal microbiome profiling and colonoscopy biopsies. Moreover, 10 patients with multiple sclerosis (as a positive control group) will undergo PET imaging and sampling for blood biomarkers. Primary outcomes include neuroinflammatory burden (biochemical and PET), longitudinal functional outcomes (Glasgow Outcome Scale Extended) and gut microbiota composition. Statistical modelling, including regression models and machine learning, will be applied to identify predictors of outcome and disease progression.

**Ethics and dissemination:**

Ethical approval has been obtained from the Ethical Committee of the Wellbeing Services County of Southwest Finland. Written informed consent will be obtained from all participants. The results will be disseminated through peer-reviewed publications, conference presentations and stakeholder engagement, with the aim of developing personalised diagnostics and novel therapeutic targets to alleviate the chronic consequences of TBI.

**Trial registration number:**

NCT06927063.

**Strengths and limitations of this study**

- This prospective observational study follows up two well-characterised, previously recruited traumatic brain injury (TBI) cohorts (TBIcare and Pathophysiological Characterisation of Severe TBI), combining their extensive acute-phase and subacute-phase biomarker and imaging data with new cross-sectional chronic-phase assessments. This enables unique longitudinal analyses of the microbiota–gut–brain axis, chronic neuroinflammation and functional recovery up to 15 years post-injury across all severities of TBI.
- Comprehensive multimodal assessments—including advanced MRI, positron emission tomography (PET) imaging, blood-based proteomics/lipidomics/metabolomics, faecal and intestinal microbiome profiling and colonoscopy biopsies—will allow identification of mechanistic links between neuroinflammation, microbiome disruption and outcome.
- The inclusion of orthopaedic trauma controls and a multiple sclerosis control group strengthens the interpretation of the results as they represent relevant comparison groups.
- Limitations include the non-randomised observational design, potential attrition of participants given the long follow-up interval and selection bias as only survivors of previous cohorts who give consent can participate.
- Some participants may decline procedures that carry potential risks (eg, colonoscopy, PET imaging), leading to incomplete data sets across modalities.

## Introduction

 Traumatic brain injuries (TBIs) are among the most serious health problems worldwide and impose a significant burden on the injured individuals, their proxies and society. It is estimated that >50 million people in the world suffer a TBI each year and that half of the people sustain a TBI in their lifetime.^1^ Despite the significant impact on health, many fundamental questions about pathophysiology, diagnostics and treatment remain poorly understood. The main reason for this is the complexity and individuality of TBI despite its initial severity, where no two injuries are identical.^1^

The key to developing effective therapies for TBI lies in better understanding and identification of the mechanisms underlying the primary and secondary pathology associated with TBI. Primary injury occurs at the time of head impact and causes direct damage to neural tissue, whereas secondary injury develops minutes to months after mechanical trauma and may progressively contribute to neurologic impairment during years after injury. There is a call for more research on the relationship between TBIs of different severities and their long-term effects and likely association with neurodegenerative diseases.^2^ In a significant minority (10%–30%) of patients with TBI, TBI has been identified as a trigger of progressive neurological deterioration and a risk factor for chronic neuroinflammation and/or subsequent neurodegenerative disease.^2^ There is increasing evidence that the immune system plays a significant role in the TBI pathogenesis. TBIs of varying severity can lead to chronic neuroinflammation, which can potentially be detected on head positron emission tomography (PET) and/or by biomarkers in peripheral blood,^3 4^ and which can persist years after TBI.^1^ A basic premise is that inflammation is associated with almost all brain injuries.^5^

Given the far-reaching influence of the gut microbiome on the central nervous system, there is also growing interest in studying the role of the microbiota–gut–brain axis in patients with TBI.^6^ The microbiota–gut–brain axis can be understood as an integrative physiological model encompassing afferent and efferent signals of neural, hormonal and immunological origin, and dysfunction of this axis has pathophysiological consequences.^6 7^ As a result, the local and systemic effects of this communication pathway on host health and disease burden are receiving increasing attention.^8^ Given the recognition that TBI is a chronic disease and a risk factor for the subsequent development of neurodegenerative diseases, and that the underlying mechanisms related to gut microbiome disruption and chronic neuroinflammation are unknown, gastrointestinal (GI) dysfunction after TBI and its impact on patient outcomes should be further investigated. A bidirectional microbiota–gut–brain axis has been proposed to be involved in the consequences of TBI. The revolving mechanism may work as shown in figure 1.^9 10^

**Figure 1:**
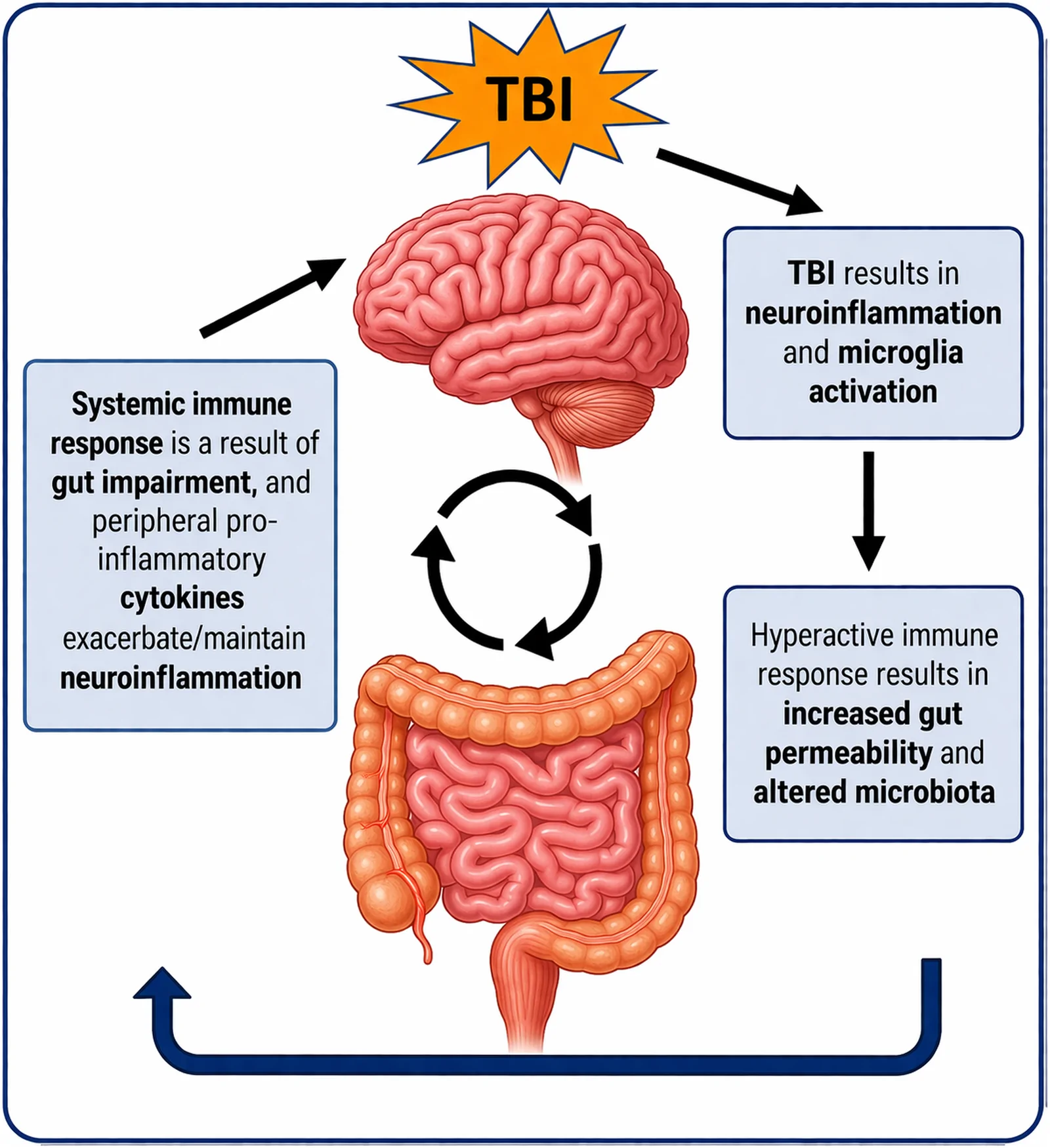
Proposed bidirectional role of microbiota–gut–brain axis in TBI. TBI, traumatic brain injury.

To clarify how these assessments fit together, we outline the conceptual framework underlying the study’s hypotheses (figure 1). TBI is proposed to disrupt intestinal barrier integrity and gut microbial composition. The resulting increase in intestinal permeability and altered microbial metabolite production promote translocation of microbial products and systemic immune activation. Through neural (vagal) and humoral signalling along the microbiota–gut–brain axis, this peripheral inflammation is hypothesised to sustain chronic neuroinflammation, which in turn contributes to progressive neurodegeneration, accelerated brain ageing and poorer long-term functional and cognitive recovery. Each node is measured directly—intestinal permeability and mucosal inflammation (colonoscopy, histology, calprotectin), microbiome and metabolites (faecal and mucosal metagenomics, blood and stool metabolomics/lipidomics), systemic immune activation (blood cytokines and chemokines), central neuroinflammation (translocator protein (TSPO) PET-MRI, blood protein biomarkers), and long-term outcome (Glasgow Outcome Scale Extended (GOSE), neuropsychological testing, advanced MRI)—allowing the proposed links to be tested along the whole pathway.

In addition to neuroinflammation, several other potential mechanisms, such as progressive axonal injury and impaired network connectivity beyond the acute phase, could contribute to the late effects of TBI; however, there is no convincing scientific evidence for many of these mechanisms when assessed at chronic time points. One reason for this is the scarcity of longitudinal studies in which subjects undergo comprehensive examinations acutely and after several years of follow-up. Previous studies that have addressed this issue show that blood levels of the biomarker neurofilament light (NfL) correlate with brain atrophy, microstructural brain injury and functional outcome in the chronic phase TBI.^11–13^ Glial fibrillary acidic protein (GFAP) and NfL can remain elevated months to years after TBI and exhibit different temporal profiles. These elevations correlate with microstructural brain injury, accelerated brain ageing and functional decline (figure 2).^13^ Identifying potential therapeutic pathways to prevent secondary brain injury and later neurodegeneration after TBI is of great importance in reducing patient morbidity. Adding the microbiota–gut–brain axis to this entity, which may influence the chronicity of neuroinflammation, may also lead to development of therapies that influence the microbiome.

**Figure 2:**
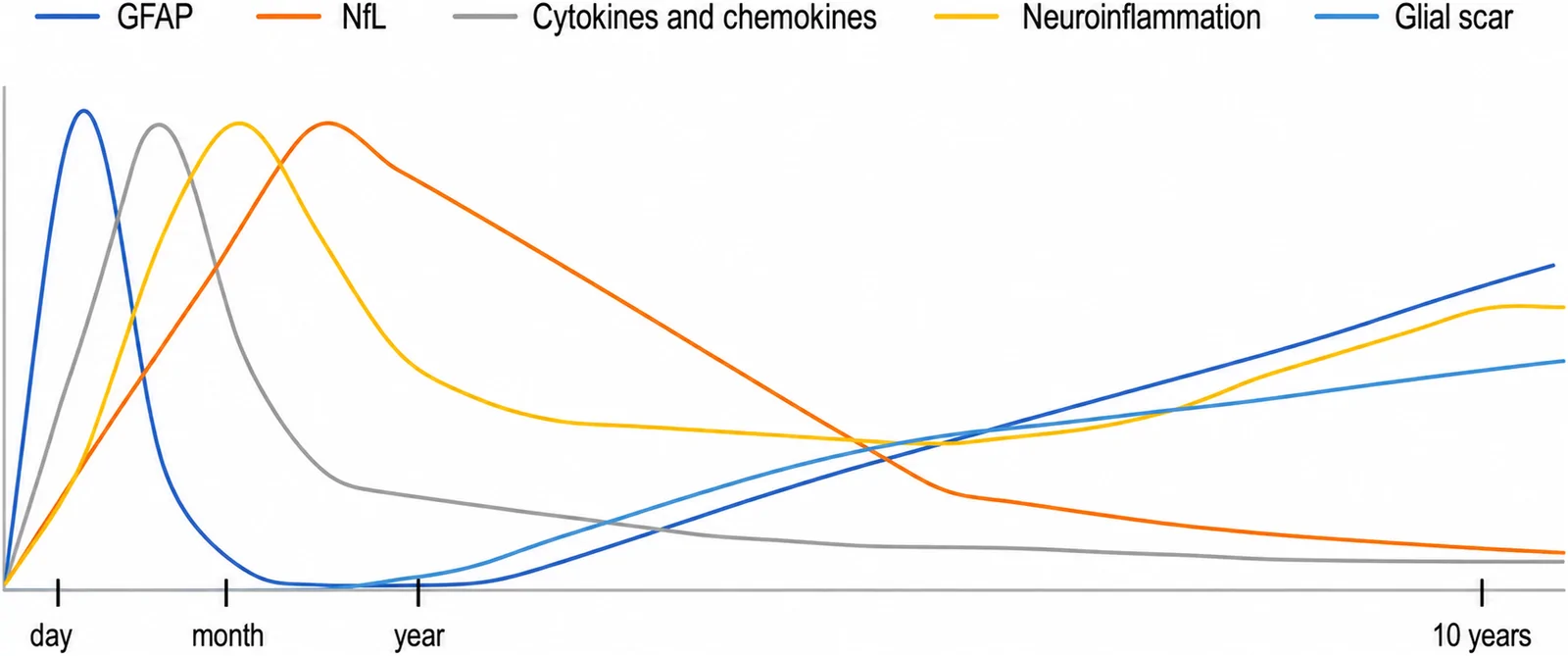
Overview of the proposed biochemical processes and blood biomarker levels in traumatic brain injury in patients who develop progressive disease. GFAP peaks at 24 hours in blood but increases again after 1 year in response to glial scarring and possibly neuroinflammation. NfL peaks between months-year in blood and decreases thereafter but remains elevated for up to several years. Pro-inflammatory and anti-inflammatory cytokines remain elevated due to chronic immune activation. Neuroinflammation involves both a protective and a damaging response, the latter of which dominates in pathology that progresses after months. GFAP, glial fibrillary acidic protein; NfL, neurofilament light.

## Methods and analysis

### Study hypothesis

The long-term recovery and microbiota–gut–brain axis disruption after TBI (OVERCOME-TBI) project includes two hypotheses:

1. Blood-based protein, metabolomic and inflammatory biomarkers can identify patients with TBI who exhibit signs of progressive neurodegeneration (progressive brain ageing, functional and/or cognitive decline) in the chronic phase of TBI.
2. Patients with progressive brain ageing, functional and cognitive decline exhibit signs of both neuroinflammation and intestinal inflammation, as well as altered gut microbiota and intestinal permeability in the chronic phase of TBI compared with (1) recovered patients and (2) control patients.

### Study overview

In the OVERCOME-TBI project, the overall goal is to collect multimodal data approximately 2–5 and 12–15 years after an earlier well-documented TBI and examine factors that influence disease progression to provide new scientific insight into the long-term pathophysiology of TBI and identify new therapeutic targets.

We will examine long-term outcome and symptom development in different severities of TBI and correlate the results with longitudinal biochemical and imaging biomarkers, and other clinical variables. We will also examine the overall neuroinflammation burden (head PET imaging, blood biomarkers), gut and faecal microbiome and metabolomic and histologic differences in the intestine between different severities of TBI and controls. Understanding the influence of the microbiota–gut–brain axis in the context of TBI may open new avenues for therapeutic approaches for TBI survivors.

### Outcome measures and expected results

#### Primary outcome measures

1. Cross-sectional and longitudinal biochemical neuroinflammation burden
  - Overall neuroinflammation burden as measured by levels of blood-based proteomics, metabolomics and lipidomics in patients with TBI compared with trauma controls at 2–5 and 12–15 years after initial measurements.
2. Longitudinal functional outcome, symptom burden, quality of life and neuropsychological performance
  - Measured with GOSE, Rivermead Post Concussion Symptoms Questionnaire (RPCSQ), Quality of Life after Brain Injury (QOLIBRI) and CANTAB.
3. Cross-sectional changes in microbiota
  - DNA from stool sample microbiome and intestinal wall microbiome assessed by deep shotgun metagenomics approach in patients with TBI compared with trauma controls.
4. Cross-sectional inflammation burden on head PET
  - Neuroinflammation measured using the [^11^C]PK11195 radioligand from whole brain and various regions of interest in patients with TBI compared with trauma controls and multiple sclerosis (MS) controls.

#### Secondary outcome measures

1. Longitudinal changes in brain white and grey matter microstructure
  - Changes in brain white and grey matter microstructure as measured with diffusion tensor metrics
2. Longitudinal brain ageing
  - Accelerated brain ageing calculated at a whole brain level using T1-weighted images and at a voxel-based level as the annualised Jacobian determinants in white matter and grey matter, referenced to a population of healthy control subjects using the Cam-CAN MRI dataset.
3. Resting state functional connectivity
  - Changes in resting-state functional network connectivity as measured with blood-oxygen-level-dependent functional MRI.

The expected research results are (1) the development of objective biochemical, microbiological and imaging methods to identify patients with progressive post-TBI brain disease and to reduce the variance in outcome prediction using novel, rich and multimodal data and (2) identification of microbiome–gut–brain connections that associate with long-term outcome of TBI, thereby revealing potential intervention targets. These findings could lead to the development of tools that promote an anti-inflammatory and regenerative immune phenotype and prevent chronic neuroinflammation. The primary and secondary outcomes listed are prespecified and registered on ClinicalTrials.gov (NCT06927063). The four primary outcome domains are treated as coprimary. Each addresses a component of the two study hypotheses, while the advanced imaging measures are secondary. The integrative cross-modal and machine-learning analyses that link these domains are exploratory and hypothesis-generating, and multiplicity across the high-dimensional, multimodal outcomes will be controlled by false discovery rate (FDR) correction, as described in the Statistics section.

### Work plan and schedule

The project includes two existing prospectively collected datasets, described in figure 3. Additionally, 10 patients with MS will be recruited from the neurological outpatient department at Turku University Hospital, where neurologists aware of the research meet these patients as part of their routine follow-up, for PET-MRI and MRI.

**Figure 3:**
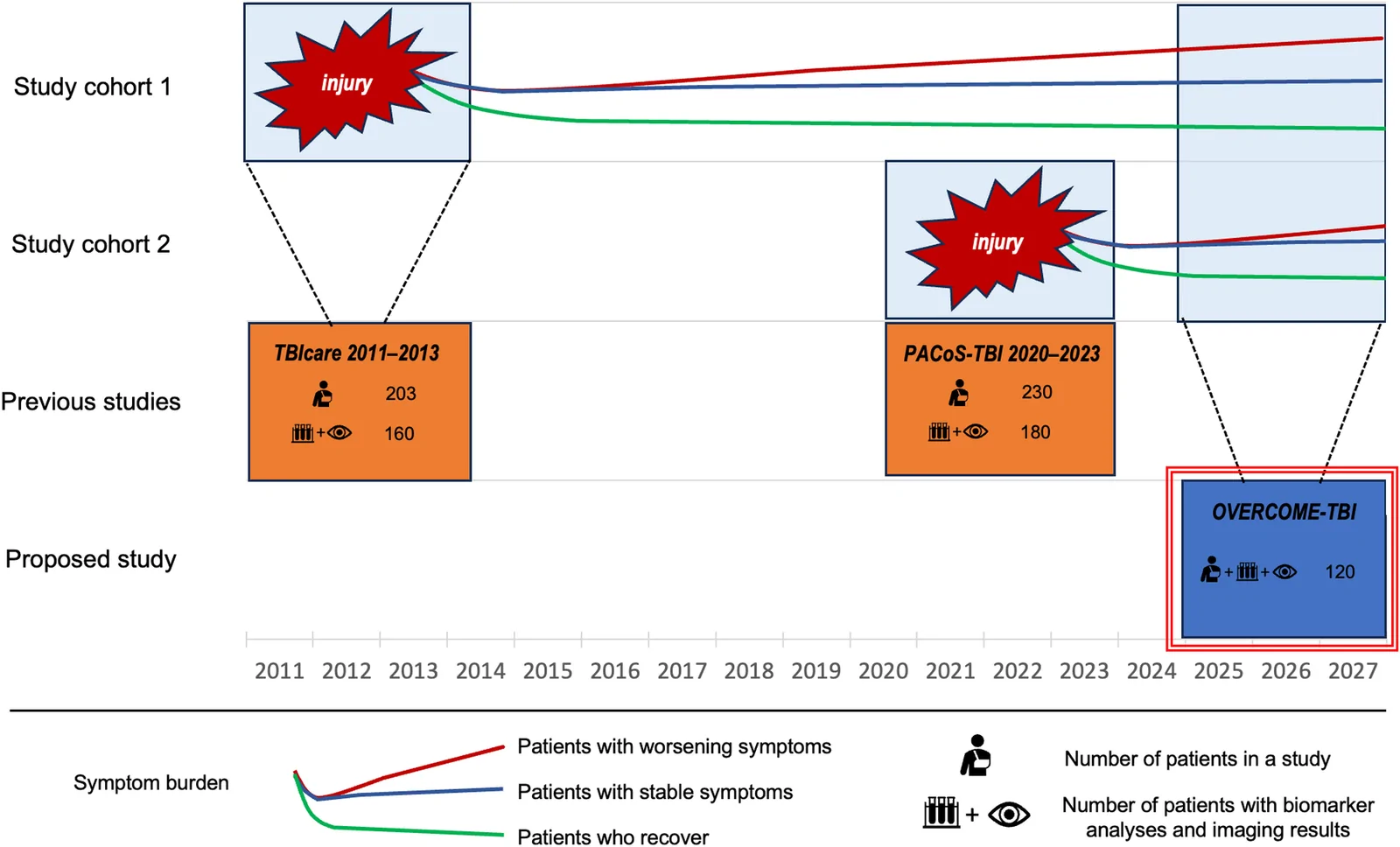
Description of the study materials. The prospective study cohorts recruited earlier (TBIcare and PACoS-TBI) provide the pool of patients with a history of TBI sustained 2–5 and 12–15 years earlier to be recruited. The coloured lines show different trajectories of recovery/symptom burden over time. The light blue boxes represent the time points studied in the studies. In the TBIcare study, biomarker levels were examined at admission, the following 7 days and at a follow-up visit approximately 9 months after injury. The TBIcare study also includes MRI scans done after injury and at the follow-up visit. The PACoS-TBI data include two daily biomarker assessments during the first 10 days after injury and at follow-up approximately 6 months after injury. The proposed study box shows an estimate of the number of patients that can be recruited. The red frame in the figure above represents the same study phase, which has the corresponding red frame in figure 5. PACoS-TBI, Pathophysiological Characterisation of Severe Traumatic Brain Injury.

The first study visit took place in October 2025, and all study visits (recruitment, imaging sessions and colonoscopies, and data collection) are planned to be completed by the end of 2027. Multimodal data analysis will begin thereafter.

### Research materials and methods

#### Study design and source cohorts

The OVERCOME-TBI project is a research project that builds on a body of previous research on two patient cohorts: (1) TBIcare (Evidence-based Diagnostic and Treatment Planning Solution for Traumatic Brain Injuries) project in which patients were recruited between 7 December 2011 and 11 November 2013 and (2) PACoS-TBI (Pathophysiological Characterisation of Severe TBI) in which patients were recruited between 1 November 2020 and 31 December 2023.

The new dataset is derived from the OVERCOME-TBI recruitment, which will provide a comprehensive analysis of the TBIcare and PACoS-TBI cohorts at the chronic time point following TBI. The organisation and content of the datasets included in OVERCOME-TBI are presented in table 1 and figure 3. The study will involve a multimodal analysis of a cohort of patients and control subjects who were prospectively assessed during the acute stage in the TBIcare and PACoS-TBI studies conducted by our research group.

**Table 1:** Variables and assessments by cohort and study phase

Domain	Variable/instrument	TBIcare (acute–subacute)	PACoS-TBI (acute–subacute)	OVERCOME-TBI (chronic)	Status
Demographics	Age, sex, education, comorbidities, medications	Yes	Yes	Yes (updated)	Longitudinally comparable
Injury characteristics	GCS, injury mechanism, CT (Marshall Helsinki CT Score), severity class	Yes	Yes	N/A (historical)	Baseline only
Functional outcome	GOSE	Yes	Yes	Yes	Longitudinally comparable
Symptoms/QoL	RPCSQ, QOLIBRI, RAND-36, BNI-FS	RPCSQ, QOLIBRI, RAND-36	RPCSQ	Yes	Longitudinally comparable (where collected before)
Neuropsychology	CANTAB (8 subsets)	Yes	No	Yes	Longitudinally comparable (TBIcare and OVERCOME-TBI)
MRI, structural	Structural T1/T2/FLAIR/3D SWI	Yes	Yes	Yes	Longitudinally mostly comparable (protocol/scan differences)
MRI, advanced	DTI, NODDI, rs-fMRI	Yes (DTI, rs-fMRI)	Yes (DTI, rs-fMRI)	Yes	Longitudinally mostly comparable (protocol/scan differences)
PET	(¹¹C)PK11195 TSPO-PET	Yes (on selected patients and controls)	No	Yes (on most patients and controls)	Longitudinally mostly comparable (protocol/scan differences)
Brain age	T1-based predicted age, Jacobian	Derived retrospectively	Derived retrospectively	Yes	New analysis
Protein BBBMs	Aβ40, Aβ42, GFAP, H-FABP, IL-10, NfL, S100B, t-tau, inflammatory mediators	Yes (excluding inflammatory mediators)	Biobanked	Sample collected at chronic visit, biobanked	Longitudinally mostly comparable (assay differences)
Blood Omics	Blood polar metabolites and lipids	Yes	Biobanked	Sample collected at chronic visit, biobanked	Longitudinally mostly comparable (assay differences)
GI variables	Rome IV (IBS), Bristol Stool Chart, diet, supplements, smoking, alcohol, travel, antibiotics, GI surgery, calprotectin	No	No	Yes	New at chronic phase
Microbiome	Faecal shotgun metagenomics; intestinal-wall microbiome (brush+biopsy)	No	No	Yes	New at chronic phase
Intestinal tissue	Colonoscopy histology, mucosal metabolomics	No	No	Yes	New at chronic phase
Follow-up schedule	Assessment time points	Acute and subacute (1–7 days), follow-up visit (~6–9 months)	Acute and subacute (1–10 days), follow-up visit (~6–9 months)	One chronic visit: 12–15 years (TBIcare)/2–5 years (PACoS-TBI)	–

Yes, assessment performed and data available for that phase; Biobanked, biological sample collected and stored at that visit, with the assay performed later; Derived retrospectively, measure computed from previously acquired data; N/A (historical), recorded only at the time of the original injury; No, not collected.

Aβ40, β-amyloid isoform 1–40; Aβ42, β-amyloid isoform 1–42; BBBM, blood-based biomarker; BNI-FS, Barrow Neurological Institute Fatigue Scale; CANTAB, Cambridge Neuropsychological Test Automated Battery; 3D SWI, 3-Dimensional susceptibility-weighted imaging; DTI, diffusion tensor imaging; FLAIR, fluid-attenuated inversion recovery; GCS, Glasgow Coma Scale; GFAP, glial fibrillary acidic protein; GI, gastrointestinal; GOSE, Glasgow Outcome Scale Extended; H-FABP, heart fatty-acid binding protein; IBS, irritable bowel syndrome; IL-10, interleukin 10; N/A, not available; NfL, neurofilament light; NODDI, neurite orientation dispersion and density imaging; PET, positron emission tomography; QoL, quality of life; QOLIBRI, Quality of Life after Brain Injury; RAND-36, RAND 36-Item Short Form Health Survey; RPCSQ, Rivermead Post-Concussion Symptoms Questionnaire; rs-fMRI, resting-state functional MRI; S100B, S100 calcium-binding protein B; TBI, traumatic brain injury; TSPO, translocator protein; t-tau, total tau.

OVERCOME-TBI is a single-centre study: recruitment, clinical assessments, imaging and sample collection take place at Turku University Hospital, whereas biomarker, omics and other specialised analyses are performed by national and international collaborating laboratories.

OVERCOME-TBI is an prospective, observational study with longitudinal follow-up data. It has two data components: (1) a prospective longitudinal component, comprising demographic, clinical, biochemical, imaging and outcome data already collected during the acute and subacute phases of the original TBIcare and PACoS-TBI studies, which enable within-participant longitudinal comparison and (2) a cross-sectional component, comprising the new chronic-phase assessments introduced by OVERCOME-TBI (chronic-phase blood biomarkers, advanced MRI, TSPO-PET, faecal and intestinal-wall microbiome profiling, colonoscopy with biopsies and repeat functional/neuropsychological testing), performed once at 2–5 and 12–15 years after injury. Longitudinal analyses link the previously collected baseline data to the chronic-phase measures; cross-sectional analyses compare patients with TBI against orthopaedic trauma and MS control groups at the chronic time points.

The overall aim is to collect multimodal data approximately 2–5 and 12–15 years after a well-documented TBI, and to investigate factors influencing the course of the disease to gain new scientific knowledge about the long-term pathophysiology of TBI and the role of neuroinflammation, as well as to identify new potential therapeutic targets. The TBIcare study included 203 patients with TBIs of all severities and 40 patients with orthopaedic injuries without TBI. The PACoS-TBI study included 253 patients with TBIs of all severities and 39 patients with orthopaedic injuries without TBI. From these cohorts, we will use demographic, biochemical and imaging data (from admission, hospital stay and follow-up phases), as well as functional and neuropsychological outcome data. To demonstrate which variables are longitudinally comparable across cohorts, and which are newly introduced at the chronic phase, a description of assessments by cohort and study phase is provided in table 1.

#### Clinical data collection and objectives

In the OVERCOME-TBI study, patients also undergo a comprehensive clinical examination. Information about their diagnosed diseases, medications taken (including duration) and any new head injuries after the initial injury is carefully recorded. Baseline data collection includes mode of delivery, underlying medical conditions, recent international travel, height and weight, special diets and use of dietary supplements, smoking status, alcohol consumption, recent antibiotic treatments, history of GI surgery and irritable bowel syndrome (IBS) symptoms (assessed using the Rome IV questionnaire). Patients are asked to describe the consistency of their stools according to the Bristol Stool Chart. These data are supplemented with the patients’ original health data collected in the TBIcare and PACoS-TBI studies.

This interview-based information is verified and, where necessary, supplemented using the electronic health records of the treating hospital system (Wellbeing Services County of Southwest Finland/Turku University Hospital, Turku, Finland). With each participant’s consent, records from the departments of neurosurgery, neurology, gastroenterology and emergency medicine are reviewed, covering subsequent healthcare contacts, incident diagnoses, medications, procedures, rehabilitation and follow-up within these specialties, thereby reducing reliance on participant recall.

We will examine (1) the long-term outcomes and symptom development across different severities of TBI, correlating findings with longitudinal investigations (MRI, PET imaging, biomarkers) and other clinical variables and (2) the gut and faecal microbiome, as well as intestinal metabolomic and histological differences between different severities of TBI and controls. Specifically, the study has the following objectives: (1) to examine longitudinal outcomes and outcome trajectories (approximately 2–5 and 12–15 years), and quality of life after TBI, (2) to examine if biochemical biomarkers during the acute and subacute stages (1–7 days and 6–9 months after injury) predict long-term neurocognitive and functional outcomes and outcome trajectories, (3) to investigate whether surrogate markers of neuroinflammation (PET imaging, biochemical biomarkers) correlate with long-term outcomes and can distinguish patients with TBI from orthopaedic controls at 2–5 and 12–15 years, (4) to examine longitudinal and long-term changes in brain microstructure, networks, and brain ageing using advanced MRI, (5) to assess associations between IBS symptoms, gut and faecal microbiome and calprotectin levels in patients with TBI and orthopaedic trauma controls, (6) to examine stool samples and endoscopically collected intestinal samples (histology, microbiota and metabolomics) in IBS-symptomatic and asymptomatic patients with TBI and orthopaedic trauma controls, (7) to examine metabolomic and lipidomic profiles in blood, stool samples and colonoscopy pinch biopsies in IBS-symptomatic and asymptomatic patients with TBI and orthopaedic trauma controls to study overall metabolomic changes in post-TBI health and (8) to examine associations between brain microstructure, networks and brain neuroinflammation (biochemical biomarkers, PET) and gut findings (microbiome, histology).

#### Study population and recruitment

From those 181+25 (patients with TBI+orthopaedic controls) patients who participated fully (=attended the outcome visit and underwent imaging) in the TBIcare study, and of those 235+39 (patients with TBI+orthopaedic controls) patients who participated fully in the PACoS-TBI study, the estimated number of those patients who will give their consent to participate in this study is 120. We will expect to be able to recruit 20 controls. It is anticipated that the entire recovery period (and the various courses of recovery) of TBI can be covered comprehensively, so 70 patients from the TBIcare study (12–15 years) and 50 patients from the PACoS-TBI study (2–5 years) will participate. It is expected that 10 orthopaedic control subjects from both studies will participate. For the PET-MRI imaging and blood-based biomarker assessment, 10 patients with MS will be additionally recruited as positive controls.

#### Inclusion and exclusion criteria

The inclusion process will be as follows (figure 5):

- Primary inclusion criteria
  - All those who are alive and give their consent will be included.
- Secondary inclusion criteria (gastroenterology)
  - All those who are alive and give their consent will be included.
- Inclusion criteria for control patients with MS
  - MS diagnosis in accordance with McDonald 2017 criteria.
  - Significant white matter lesion load (a minimum of 10 white matter lesions).
  - Secondary progressive MS.
- Primary exclusion criteria
  - For patients with TBI there are no exclusion criteria, and all eligible patients who give consent will be recruited. Those patients and controls with contraindication for head MRI (MRI-incompatible heart pacemaker, weight over 200 kg, mechanical heart valve prosthesis, first trimester of pregnancy, orbital area tattoo) will not undergo head MRI. Instead, they will undergo the other assessments. For controls, those who have suffered a TBI or any other brain disorder after the TBIcare or PACoS-TBI study will be excluded.
- Secondary exclusion criteria (gastroenterology)
  - Diagnosis of IBD or other diagnosis besides IBS causing severe GI symptoms, such as microscopic colitis or bile acid diarrhoea.
  - Poorly controlled coeliac disease.
  - Colorectal cancer diagnosed within 5 years.
  - Antibiotic or probiotic treatment, ongoing or previous month.
  - Unwilling or unable to undergo colonoscopy and/or standard bowel preparation.
  - Significantly increased risk of heart or kidney failure or electrolyte imbalances due to bowel preparation (frailty, serious pre-existing heart or kidney insufficiency).
  - Altered bowel anatomy after significant operation. Appendicectomy or cholecystectomy are considered minor operations.
- Exclusion criteria for patients and controls undergoing PET-MRI imaging
  - Patients with other neurodegenerative disease than MS.
  - Patients with history of TBI necessitating hospital admission.
  - Patients with other autoimmune disease than MS.
  - Patients with other significant or malignant underlying disease of any other organ system.
  - Patients that are pregnant or breastfeeding.
  - Corticosteroid treatment within 4 weeks of imaging.
  - Patients with claustrophobia, or a history of moderate to severe anxiety disorder or panic attacks (which could potentially lead to preterm termination of the imaging).
  - Contraindication to PET scan investigations.
  - Exposure to experimental radiation in the past 12 months such that radiodosimetry limits would be exceeded by participating in this study.
  - Intolerance to previous PET scans, that is, previous hypersensitivity reactions to any PET ligand ([^11^C]PK11195 radioligand) or imaging agent or failure to participate in and comply with previous PET scans.

As for neurological and cognitive examination, withdrawals are unlikely. It is expected that not all patients are willing to undergo colonoscopy and histological sampling. However, they may be included in neurological and faecal microbiome examinations. There will be no replacement for any dropouts. Data collected prior to dropout will be used.

The patients will undergo neurological and cognitive testing, blood sampling for blood-based biomarker analyses, head MRI, colonoscopy. Up to 60 patients with TBI will also undergo PET imaging (radioligand [^11^C]PK11195). Patients with orthopaedic injuries will serve as controls and will undergo similar assessments (figures 4 and 5). For PET-MRI, control patients include patients with orthopaedic injuries from the TBIcare and PACoS-TBI cohorts (up to 10) and patients with MS (up to 10).

**Figure 4:**
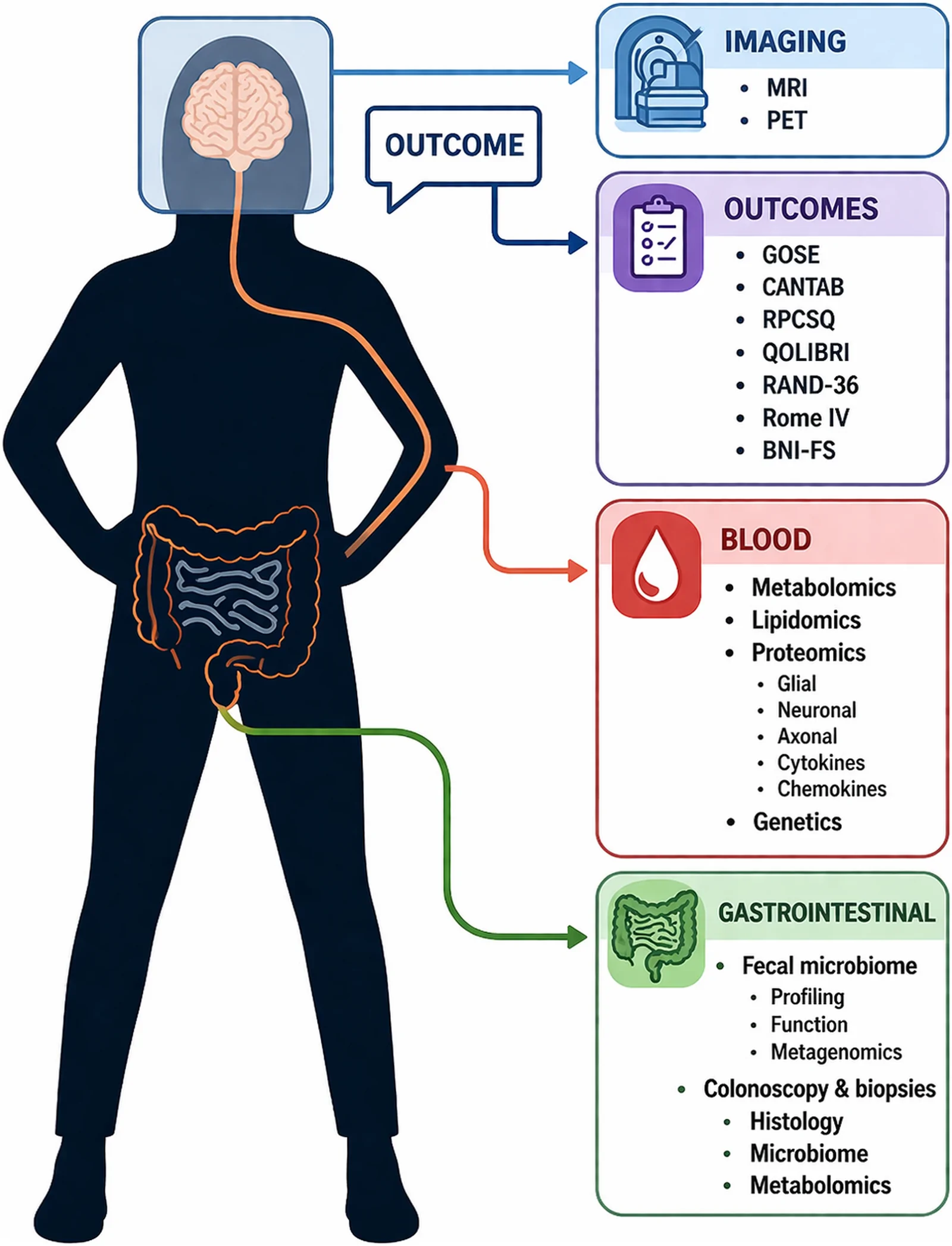
Description of the study assessments included in the clinical (neuro and gastro) and gastroenterological visits. See body text for the description of the assessments. BNI-FS, Barrow Neurological Institute Fatigue Scale; CANTAB, Cambridge Neuropsychological Test Automated Battery; GOSE, Glasgow Outcome Scale Extended; PET, positron emission tomography; QOLIBRI, Quality of Life after Brain Injury; RPCSQ, Rivermead Post Concussion Symptoms Questionnaire.

**Figure 5:**
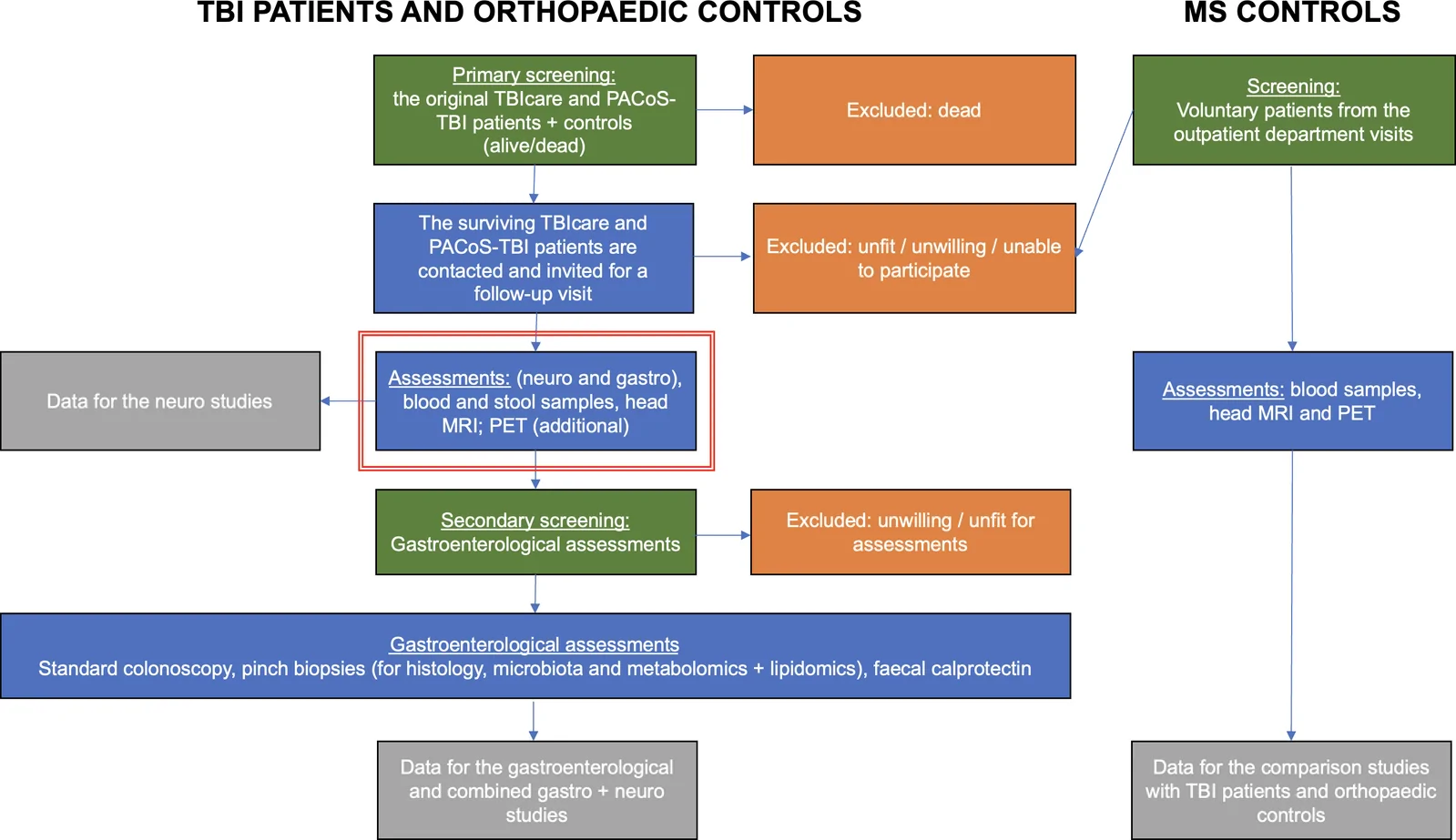
OVERCOME-TBI study flow chart. The red frame in the figure above represents the same study phase, which has the red frame in figure 3. MS, multiple sclerosis; PACoS-TBI, Pathophysiological Characterisation of Severe Traumatic Brain Injury; PET, positron emission tomography.

#### Clinical, neuropsychological and MRI assessments

The clinical assessments of the OVERCOME-TBI will include the same evaluation tools that were used in the original TBIcare and PACoS-TBI studies: (1) GOSE, (2) RPCSQ, (3) QOLIBRI, (4) RAND-36 (general health questionnaire), (5) gastroenterological questionnaire panel including the Rome IV Diagnostic Questionnaire and (6) BNI-FS (Barrow Neurological Institute Fatigue Scale) (figure 4). Cognition of the participating subjects will be tested using the Cambridge Neuropsychological Test Automated Battery (CANTAB) computerised testing battery (same eight subtests as in TBIcare).

Structural and advanced MRI will be performed on a 3.0T GE Signa Premier scanner (GE HealthCare, Milwaukee, Wisconsin, USA) at Turku University Hospital (online supplemental appendix). The imaging protocol includes the following sequences: (1) three-dimensional (3D) sagittal T1-weighted magnetisation-prepared rapid gradient echo (MPRAGE; 0.5 mm isotropic voxels, TI 900 ms, flip angle 8°), (2) 3D sagittal T2-weighted fluid-attenuated inversion recovery (FLAIR; Cube acquisition, 0.5 mm isotropic voxels, TR/TE/TI 6000/105/1768 ms, echo train length 200, HyperSense acceleration factor 1.4), (3) axial T2-weighted fast spin echo (FSE; 4.0 mm slice thickness with 0.4 mm gap, 36 slices, TR/TE 7200/89 ms, matrix 384×320, FOV 24 cm), (4) 3D axial susceptibility-weighted angiography (SWI; 1.0 mm isotropic voxels, 7 echoes, TE 25 ms, flip angle 15°), (5) multishell diffusion tensor imaging acquired in three separate runs with b-values of 1000, 1500, and 2800 s/mm² and 9, 30 and 64 non-collinear diffusion-encoding directions, respectively (2.0 mm isotropic voxels, 79 slices, TR/TE 6200/72 ms, matrix 128×128, FOV 24 cm, anterior-to-posterior phase encoding, 3 non-diffusion-weighted volumes per shell, HyperBand simultaneous multislice factor 2) and (6) resting-state blood-oxygen-level-dependent (BOLD) functional MRI (gradient echo EPI, 3.4 mm isotropic voxels, 48 slices, TR/TE 920/30 ms, flip angle 70°, 900 volumes, 5 dummy acquisitions, interleaved slice order, HyperBand simultaneous multislice factor 4). Axial sequences are angulated parallel to the anterior commissure-posterior commissure line. Participants undergoing resting-state fMRI are instructed to keep their eyes open and remain still without focused thought. Subjects who undergo PET-MRI will be scanned on the integrated GE Signa PET-MRI 3.0T system as described below. The multishell diffusion data will be used to derive conventional diffusion tensor metrics (fractional anisotropy, mean diffusivity, axial diffusivity and radial diffusivity) as well as neurite orientation dispersion and density imaging parameters, including the neurite density index and orientation dispersion index. Multishell constrained spherical deconvolution-based tractography will be performed to assess structural connectivity. Resting-state fMRI data will be preprocessed using standard pipelines including motion correction, spatial normalisation, temporal filtering and nuisance regression, and analysed to evaluate functional connectivity between predefined brain networks. Accelerated brain ageing will be calculated at a whole brain level as the predicted age difference defined using T1-weighted images, and at a voxel-based level as the annualised Jacobian determinants in white matter and grey matter, referenced to a population of over 600 healthy control subjects, which is an earlier collected dataset entitled Cam-CAN data repository.^13 14^

#### PET imaging of neuroinflammation

Neuroinflammation will be assessed using TSPO-PET–MRI imaging. TSPO is an 18 kDa mitochondrial membrane protein that is upregulated in activated microglia and other glial cells in response to neuroinflammation. Before a PET scan using PET-MRI, a brief MRI scan is performed solely for alignment purposes. PET imaging will be performed using the TSPO radioligand [^¹¹^C]PK11195, which binds to TSPO and enables in vivo quantification of neuroinflammatory activity. Imaging will be conducted on a GE Signa PET–MRI scanner (GE Healthcare, Milwaukee, Wisconsin, USA). The target radioactivity dose of the administered [^11^C]PK11195 radioligand is 500 MBq. 60-minute dynamic imaging is started simultaneously with the intravenous, smooth bolus injection of the radioligand. Head movements during the scan are minimised using support around the head. Image reconstruction is performed using 17 time frames (2×15, 3×30, 3×60, 7×300 and 2×600 s; total, 3600 s) as described previously.^15^ The dynamic data is then smoothed using a Gaussian 2.5 mm postreconstruction filter.^16^ The possible displacements between frames are corrected using mutual information realignment in SPM12. Finally, all the PET images are resliced to match 1 mm MR voxel size. For the estimation of [^11^C]PK11195 distribution volume ratio (DVR), the time–activity curve corresponding to a reference region devoid of specific TSPO binding is acquired for each PET session using a supervised cluster algorithm approach with four predefined kinetic tissue classes (SuperPK software, SCA4 classification)^17 18^ as described previously.^19^ As an additional step, the intersection of the clustered reference region maps of each session is used as a common clustered reference region. Regional DVRs are estimated by using reference tissue input Logan method,^20^ with time interval from 20 to 60 min.

#### Biospecimen collection and biobanking

A 10 mL blood sample from the antecubital vein will be drawn into one plasma and one serum tube (=20 mL altogether) and will be divided in aliquots. The biological component of the study includes the collection and storage of peripheral blood, stool samples and intestinal biopsies. The blood is collected and processed into serum and plasma. Aliquots are stored at −70°C under biobank conditions with standardised protocols to ensure consistency. Stool samples are taken for microbiome analysis and biopsies, if performed, are stored at −70°C. All samples are pseudonymised and tracked using unique identifiers.

#### Blood-based biomarker analyses

Sample analyses are performed in several specialised laboratories, including the University of Turku (Finland), the University of Geneva (Switzerland) and Örebro University (Sweden), but because the development of analytical platforms is rapid, analyses may also be performed in several other laboratories of our research group’s collaborators. Standard operating procedures will be developed for blood biomarker assays, microbiome profiling and inflammatory cytokine analysis. Cross-laboratory calibration and quality control measures are instituted to ensure reproducibility and cross-site comparability. Blood-based protein biomarker analyses will include measurement of GFAP, NfL, S100 calcium-binding protein B, brain-derived tau, ubiquitin C-terminal hydrolase-L1 and targeted cytokines and chemokines.

Metabolomic analyses of blood and stool samples will use state-of-the-art liquid chromatography coupled to high-resolution mass spectrometry methods. Samples will be processed with our newly developed and validated monophasic extraction protocol using extensive isotope-labelled internal standards, enabling robust quantitation across a wide polarity range and high-throughput analysis of clinical samples.^21^ Multiple analytical protocols will cover polar metabolites (including microbial metabolites and neurometabolites) and lipids (lipidomics).

Global metabolomics will quantify key compound classes, including bile acids (with microbially conjugated species), free fatty acids, amino acids and a wide range of microbially derived metabolites. Neurometabolite panels will include N-acyl amides including endocannabinoids, classical neurotransmitters and their metabolites. An internal library of >1800 authentic standards, enriched for gut microbiota-derived metabolites (indole derivatives, phenolics, short-chain fatty acids, secondary bile acids, sulphated compounds), supports confident annotation. Lipidomics will use class-specific internal standards and external calibrants, covering >800 lipids.

For non-targeted identification, raw data from exposure experiments will be interrogated using molecular networking (GNPS),^22^ in silico fragmentation (MetFrag),^23^ biotransformation prediction (BioTransformer)^24^ and literature mining. Novel machine learning workflows will facilitate annotation of microbially derived metabolites.^25^ MicrobeMASST,^26^ a curated database of >60 000 microbial monocultures, will link MS/MS spectra to candidate microbial producers. MZmine^27^ will be used for data processing.

#### Faecal microbiome analyses

The faecal microbiome assessments will be done on the stool samples collected at the first visit. The patients will receive two kits: OmnigeneGUT and OmnimetGUT (DNA GenoteK, Ottawa, Ontario, Canada) for genetic and metabolomics analyses, respectively, with sampling instructions. The patients will be given the opportunity not to take part in the faecal genetic analysis. For microbiome (both taxonomical profiling and function) analysis, DNA will be extracted from one aliquot of the collected stool samples by using Powerbeads and TissueLyser II (Qiagen, Germany) and Chemagic DNA Stool 200 Kit (PerkinElmer, Massachusetts, USA) on a Chemagic 360 machine in 96-well format.^28 29^ To analyse the DNA from faecal microbiome, we aim at a deep shotgun metagenomics approach, (>6 Gb) which guarantees high coverage and depth for strain-level resolution, critical for studying transmission patterns from donor to patient. As a basis for the development of these methods for standard analyses of gut microbiome composition, diversity and function are available.^30 31^ In the faecal and intestinal omics analyses, both targeted and non-targeted metabolomics with mass spectrometric techniques coupled with chromatography will be used.

Stool (faecal) and intestinal wall microbiome samples are both processed and sequenced at the Department of Clinical Microbiology, Turku University Hospital. DNA extraction will be performed using optimal methods for each sample material. Sequencing and bioinformatics pipelines will be harmonised to ensure comparability for all sample material. The two compartments are analysed as separate datasets and then compared within this common framework.

#### Gastroenterological investigations

We expect 90% of patients to be included in the gastroenterological investigations. However, it is assumed that a stool sample will be obtained from all participants. Two factors support the expected participation. First, most patients in these cohorts have a long-standing connection with the Neurocentre at Turku University Hospital. Second, the risks, burden and potential benefits of colonoscopy and biopsies, including the detection and immediate treatment of incidental findings such as advanced polyps, are explained in the patient information sheet before consent and have been approved by the regional ethics committee. Because consent is modular, participants who decline colonoscopy still provide a stool sample and undergo the other assessments, so faecal microbiome data are expected from nearly all participants; incomplete uptake of invasive procedures is also addressed through uptake weighting and sensitivity analyses (see Statistics).

All consenting patients and orthopaedic controls included in the second screening (figure 5) will undergo standard colonoscopy, including pinch biopsies from the bowel wall at several levels (terminal ileum, ascending colon, descending colon and rectosigmoid) for histology, microbiome analysis and metabolomics. Microbiome samples will also be collected from the colonic wall using a GI microbiology brush (Hobbs Medical, Connecticut, USA). The colonic mucosa consists of an inner and an outer mucus layer.^32 33^ The inner layer is generally considered nearly sterile in a healthy colon, whereas the outer layer is rich in bacterial communities. Under certain inflammatory conditions, this homeostasis may be disrupted.^34^ The microbial composition of samples collected using the microbiology brush may therefore differ from that obtained through pinch biopsies. We will investigate the inflammatory state and changes in intestinal permeability to assess the overall inflammatory burden, together with faecal microbiome profile, blood biomarkers and head imaging (PET), and correlate the findings with neurological disease progression.

### Study risk management

Because the project includes material from two clinical studies, the following risks exist (in descending order of likelihood): (1) The number of patients in TBIcare and PACoS-TBI remains too small to answer all hypotheses—the risk exists because reliable performance calculations cannot be made in this kind of setting; the solution to this problem is that we are part of the global InTBIr consortium (https://intbir.incf.org), so it is possible to initiate a larger follow-up study, (2) inefficient use and/or pooling of a wealth of data, (3) failure to synchronise work input from different collaborators and (4) due to the highly complex brain pathophysiology of TBI, it may be that the causalities behind obtained results remain uncertain and will require further studies. The overall likelihood of risks is low.

### Statistics

#### Sample size: overall study

This is a prospective, observational study with longitudinal follow-up and a cross-sectional chronic-phase component. Because it uses a real-life approach, the data will not be collected in such a way that the groups are balanced in terms of demographic variables. The maximum number of subjects recruited, as mentioned above, is as follows: 160 patients with TBI, 30 patients with orthopaedic injuries and 10 patients with MS.

#### Sample size: PET imaging

The main target population is a TBI cohort, but we have included two other separate groups as control groups. The MS cohort (n=10) serves as a positive control group for the study. TSPO-PET studies in MS patients have shown the most robust results in the field of TSPO-PET imaging, both in terms of increased TSPO signals in the brain^19^ and in terms of association with later progression.^35 36^ The inclusion of a separate positive control cohort of MS patients in the study therefore allows direct comparison of the currently investigated patient group in which the outcome is not yet known (patients with TBI) with a patient group in which an increase in innate immune cell activation in the brain is generally known to be detectable with TSPO-PET. Imaging all patient groups with the same PET scanner and analytical methods provides the most reliable comparison for the assessment of potentially increased TSPO binding also in the TBI cohort. In addition, we included a negative control group of patients with orthopaedic injury (n=10) with otherwise known normal brain MRI findings. In a healthy brain, TSPO–PET typically shows low and uniform uptake in most brain regions. This pattern reflects the minimal expression of TSPO under normal physiological conditions, as TSPO is primarily upregulated in response to neuroinflammation.^37 38^ Based on previous experience with PET ligands,^17 19^ it is estimated that 10 subjects per group are sufficient to detect 15% difference in [^11^C]PK11195 uptake between the study groups with 90% power at a p<0.05.

### Statistical plan and analysis

#### Analysis populations and outcomes

All consenting participants from the earlier studied cohorts (TBIcare 2011–2013; PACoS-TBI 2020–2023) will be analysed at chronic time points 2–5 and 12–15 years after injury. Orthopaedic trauma and MS controls will be analysed per planned modality.

The primary outcomes are (1) neuroinflammatory burden (blood-based biomarker panels and [^11^C]PK11195 PET), (2) long-term functional outcome (GOSE) and (3) gut microbiota composition. MRI microstructure and brain-age measures are key secondary outcomes.

#### Descriptive statistics

Baseline characteristics will be summarised using means (SD), medians (IQR) and proportions, stratified by cohort, group, and time window. Standard parametric or non-parametric tests will be used for exploratory comparisons.

#### Primary analyses

- Blood biomarkers: Cross-sectional differences between TBI and orthopaedic controls will use multivariable linear models (log-transformed data), adjusting for age, sex, cohort and time since injury.
- PET: regions of interest-brain and whole-brain DVR values will be compared between groups with linear models adjusted for age, sex and cohort. Associations between PET and blood biomarkers will be assessed using partial correlations and regression.
- Outcome: Longitudinal outcomes will be analysed with mixed-effects ordinal logistic regression including age, sex, severity indicators, cohort and time window as fixed effects.
- Microbiome: Diversities will be analysed with adjusted linear models and PERMANOVA. Differential abundance will use a composition-aware method with FDR control. Associations with biomarkers and GOSE will be examined using multivariable regression. These models will be adjusted for key microbiome confounders recorded at sampling: diet, dietary supplements, smoking, alcohol use, recent travel and recent medication (including antibiotics). Recent antibiotic or probiotic use will additionally be addressed in the sensitivity analyses described below. Rome IV-defined IBS symptoms are treated as a phenotype of interest and as a stratifying subgroup variable (IBS-symptomatic vs asymptomatic) for microbiome, histology and metabolomic comparisons, and are also explored as a potential effect modifier in the gut–brain analyses. They are not treated as a confounder to be adjusted away.

#### Secondary analyses

MRI microstructure and brain-age measures will be analysed using linear models adjusted for demographic and scanner effects. These variables will also be explored in relation to PET, biomarkers and GOSE.

#### Multimodal data integration and machine learning

Because the datasets are highly multimodal while the sample size is relatively modest given the sizes of the original cohorts, machine-learning analyses are regarded as exploratory and hypothesis-generating. To keep the analyses robust, high-dimensional data will be reduced before modelling, using established dimensionality-reduction and regularisation techniques, and models will be kept as simple as the data allow. Predictive performance will be evaluated using cross-validation to limit overfitting, and results will be interpreted cautiously and, where possible, checked in the second cohort or in external data through the InTBIR consortium. The detailed analysis plan, including the specific methods for feature selection, dimensionality reduction and validation, will be prespecified for each substudy in collaboration with a statistician and systems biologists and adapted to the data and research question.

Specialised analytical and machine-learning expertise will be engaged for each analysis as the relevant work begins, drawing on the study team’s broad national and international collaborative network.

#### Multiplicity and missing data

For high-dimensional omics and microbiome analyses, Benjamini-Hochberg FDR at q=0.05 will be applied. For PET and advanced MRI region of interest analyses, FDR correction will be applied across predefined region of interest. Missing data will be addressed using multiple imputation under missing at random assumptions. Complete-case analyses will be used as sensitivity checks.

#### Bias and sensitivity analyses

To address selection and survivorship bias, baseline characteristics of participants and non-participants will be compared, cohort indicators included in all models, and uptake weights applied to modality-specific analyses. Sensitivity analyses will include exclusions for recent antibiotics/probiotics, stratification by time window and scanner/centre adjustments.

#### Model diagnostics and software

Model assumptions will be checked using standard diagnostics. Results will be reported as adjusted effect estimates with 95% CIs and q-values where relevant. Statistical analyses will be performed in RStudio (Posit PBC) and MATLAB (MathWorks, Massachusetts, USA), and analysis code will be made openly available alongside the publication.

### Data handling and record keeping

#### Case report forms

An electronic case report form will be filled for each patient, including all questionnaires used as well as thorough medical history and information about imaging, blood sampling and cognitive testing.

#### Blood, faecal and tissue samples

Blood samples will be stored in a –70°C freezer with pseudonymised identifiers. Faecal and intestinal tissue samples will be stored in a deep freeze –70°C in the Department of Microbiology at the University of Turku.

#### Data management

The case report forms will be stored at the research office in a locked cupboard. All case report forms will be de-identified and stored using a study-specific REDCap form. The electronic records will be stored at the hospital’s server (REDCap database at University of Turku), secured by a username and password. To give access for the data also for researchers outside the research group, the data will be transferred to an anonymised database, which can be given based on data sharing agreement.

The study analyses will be performed by collaborating laboratories in Finland and abroad, which requires transfer of biological samples and de-identified data. Because analytical platforms and collaborations evolve over the course of the study, the participating laboratories may change. In all cases, transfer of biological material takes place under a Material Transfer Agreement, and transfer of personal data under a Data Transfer/Processing Agreement, agreed before any sample or dataset leaves the study centre. All shared data are pseudonymised or anonymised, and microbiome and other omics data are handled in accordance with the European Union (EU) General Data Protection Regulation and applicable Finnish legislation; transfers outside the EU/European Economic Area rely on an adequacy decision or other appropriate safeguards. Samples are stored under standardised biobank conditions, with blood, faecal and intestinal samples held under the governance of the research group itself and Turku Clinical Microbiome Bank, and external access to samples or data is granted only under the corresponding agreements.

#### Study subject register

The study register will be built according to current EU data protection legislation.

### Ethics and dissemination

#### Ethics review and study registration

The ethical review board of the Wellbeing Services County of Southwest Finland approved the study protocol (VARHA/20776/13.02.02/2024). The protocol also received institutional approval from the Wellbeing Services County of Southwest Finland (T2763_2024). The protocol has also been registered to ClinicalTrials.gov (ClinicalTrials.gov identifier: NCT06927063) prior to the start of the recruitment. The protocol synopsis can be found at ClinicalTrials.gov.

#### Ethics

As described above, the study has received ethical and organisational approvals. The study will consist mainly of non-interventional assessments that do not bear special ethical concerns. The risk of serious complications, including bowel wall perforation, is less than 1/1000 for diagnostic colonoscopy.^39^ General anaesthesia is not required, but intravenous sedation and pain relief will be provided if requested by the patient. We expect these colonoscopies to reveal several important incidental findings (mostly advanced colonic polyps) that can be treated immediately and are likely to benefit patients. Bleeding risks involved with pinch biopsies are considered minimal. Therefore, discontinuation of anticoagulant medication is not recommended in the current clinical practice.

For the PET-imaging part, the participants will receive one [^11^C]PK11195 injection. The total effective dose is 2.4 mSv, which is equivalent to the average radiation dose a Finn receives in 5 months. Based on this, those patients involved in the research will not be exposed to a significantly harmful radiation burden, and therefore from the point of view of radiation biology the study can also be considered ethically acceptable. Furthermore, the treatment of the patients will not be affected by the study. The research methods are PET-MRI and MRI scans and blood sampling, which cause patients some degree of discomfort (venous cannulation, sacrifice of their time), but are not dangerous. Participation in the research is completely voluntary, and subjects may at any time suspend the study.

All subjects will be able to give informed consent and the examinations performed will not pose any health risks to the subjects. The principles of the Declaration of Helsinki will be followed.

#### Subject information and informed consent

A written information sheet about the study will be sent to all potentially eligible subjects, together with an informed consent sheet. The subjects can ask additional questions about the study before giving their consent. In case there is any doubt about the subject’s ability to understand the study and give consent, the same information will be given to the proxy.

The original TBIcare and PACoS-TBI studies obtained informed consent for those studies only; consent did not extend to participation in OVERCOME-TBI. Accordingly, new, study-specific written informed consent is obtained from every OVERCOME-TBI participant at the first study appointment. Eligible survivors from the two cohorts are re-contacted by letter—an approach approved by the regional ethics committee as part of the OVERCOME-TBI protocol (VARHA/20776/13.02.02/2024)—explaining the reason for re-contact, the purpose of the study, and the procedures involved. Interested individuals then receive the full information sheet and consent form and can ask questions from the investigators before consenting.

Consent covers all OVERCOME-TBI procedures, including clinical and neuropsychological assessment, blood sampling and blood-based biomarker and omics analyses, advanced MRI, [¹¹C]PK11195 TSPO-PET imaging, stool collection and faecal microbiome sequencing and colonoscopy with intestinal biopsies. Consent is modular: participants may agree to some assessments and decline others (eg, decline colonoscopy or PET imaging while still undergoing clinical testing, blood sampling and faecal microbiome analysis) and data collected before any withdrawal are retained. Consent also covers pseudonymised storage of samples and data, and the sharing of de-identified data with national and international collaborators for the planned analyses, under the relevant data-sharing agreements and in accordance with EU data-protection legislation and the Declaration of Helsinki.

#### Study report and publications

This study will produce multiple original publications because of the nature of the data and the possibility of comparison with results obtained from the same individuals at different time points after TBI. The focus will be on (1) the long-term outcome of TBI and its prognostic indicators and (2) the altered gut microbiota and intestinal permeability after TBI. The results of this study may reveal potential targets for reducing the inflammatory disturbance of the gut-brain axis and thus to reduce its deleterious effects on the brain.

#### Patient and public involvement

Patients and the public were not involved in the design, conduct, reporting or dissemination plans of this study. The study is based on long-term follow-up of existing cohorts and complex multimodal assessments designed by the research team. Results will be disseminated through scientific publications and conferences.

Patients and the public will, however, be engaged in dissemination through accessible channels, including public lectures, social media, podcasts and newspaper articles aimed at raising awareness of the consequences and prevention of TBI.

## Discussion

TBI diagnostics has lagged significantly behind that of many other diseases. The current methods for assessing severity and predicting outcomes are based only on initial stage variables, and there are no objective tools for monitoring the progression of the disease. While biomarkers and imaging techniques have provided valuable insights in the acute phase, the mechanisms linking chronic neuroinflammation, structural brain changes, and gut microbiota disruption to long-term outcomes remain poorly understood. This study is designed to address this gap.

By using two well-characterised prospective cohorts with detailed acute and subacute data, the OVERCOME-TBI study provides a unique opportunity to investigate the trajectory of recovery and deterioration at chronic time points up to 15 years post-injury. The multimodal approach—including PET-MRI, advanced structural imaging, proteomics, lipidomics, metabolomics, faecal metagenomics and gut biopsies—will allow investigation of how inflammatory processes in the brain and gut interact and contribute to long-term disability. The inclusion of orthopaedic trauma and MS control groups strengthens interpretability by allowing comparison with both non-brain injury trauma and disease with established neuroinflammatory pathology.

Anticipated contributions of the study include identifying biological and imaging markers of progressive neurodegeneration, clarifying the role of gut dysbiosis and intestinal permeability in chronic TBI sequelae, and developing integrative models linking neuroinflammation to functional and cognitive outcomes.

Several limitations must be acknowledged. The non-randomised observational design precludes causal inference, and participation is limited to survivors of the original cohorts who agreed to long-term follow-up, leading to survival and selection bias. Attrition is to be expected during the long follow-up period, and the uptake of invasive procedures such as PET imaging and colonoscopy may be incomplete, resulting in variable completeness of data across modalities. In addition, PET has limited specificity and genetic polymorphism effects, while the composition of the microbiome is influenced by environmental and dietary factors that may confound the associations.

The risk–benefit ratio of the study is favourable. Radiation exposure from PET-MRI is low and within accepted research limits, and colonoscopy carries a very low risk of complications when performed according to standard protocols. All participants give informed consent and can decline individual procedures.

In summary, OVERCOME-TBI will generate one of the most comprehensive longitudinal datasets on chronic TBI, integrating systemic, neuroimaging and microbiome markers. It is expected that this work will improve the mechanistic understanding of long-term disability after TBI and provide information on prognostic and therapeutic strategies.

## Supplementary material

10.1136/bmjopen-2026-120552 online supplemental appendix 1
