# Supplementary material for: Long-term recovery and microbiota–gut–brain axis disruption after traumatic brain injury (OVERCOME-TBI): protocol for a prospective, observational clinical study
Source: BMJ Open. 2026 Sep 23;16(9):e120552. doi: 10.1136/bmjopen-2026-120552 (PMC13630022; doi:10.1136/bmjopen-2026-120552)
Supplement: Supplementary file 1 [file bmjopen-2026-120552.reviewer_comments.pdf]

PEER REVIEW HISTORY

BMJ Open publishes all reviews undertaken for accepted manuscripts. Reviewers are asked to complete a checklist review form and are provided with free text boxes to elaborate on their assessment. These free text comments are reproduced below.

ARTICLE DETAILS

Title (Provisional)

Long-term recovery and microbiota-gut-brain axis disruption after traumatic brain injury (OVERCOME-TBI) – Protocol for a prospective, observational clinical study

Authors

Artosalo, Matti; Östberg, Anna; Tenovuo, Olli; Roine, Timo; Mohammadian, Mehrbod; Kallonen, Teemu; Salminen, Kimmo; Stenholm, Teppo; Hakanen, Antti; Sainio, Teija; Hirvonen, Jussi; Dickens, Alex M; Orešić, Matej; Büki, András; Hyotylainen, Tuulia; Menon, David; Newcombe, Virginia; Chiollaz, Anne-Cécile; Sanchez, Jean-Charles; Mondello, Stefania; Airas, Laura; Posti, Jussi P.

VERSION 1 - REVIEW

**Reviewer**	**1**
**Name**	**Pongpirul, Krit**
**Affiliation**	**Chulalongkorn University Faculty of Medicine, Preventive and Social Medicine**
**Date**	**27-Jun-2026**
**COI**	**None**

Major Comments:

1. Although OVERCOME-TBI is described as a prospective observational study, it appears to represent a long-term follow-up study based on two previously established prospective cohorts (TBIcare and PACoS-TBI), with additional cross-sectional assessments performed at chronic time points. It would be helpful to explicitly state the study design to facilitate readers' understanding of the analytical framework and interpretation of the findings.

2. Given that the present study integrates two cohorts recruited during different periods, a summary table showing variables collected in each phase would improve transparency. This could include demographics, injury characteristics, functional outcomes, neuropsychological assessments, imaging modalities, biomarkers, gastrointestinal variables, microbiome analyses, and follow-up schedules. Such a table would help readers understand which variables are longitudinally comparable and which assessments are newly introduced.

3. The protocol would benefit from explicitly stating whether the original studies permitted future re-contact, long-term follow-up, and secondary analyses. Clarification on whether new informed consent will be obtained for all additional procedures, including PET imaging, colonoscopy, microbiome sequencing, and international collaborative analyses, would also be helpful.

4. Finland has a major advantage in terms of national healthcare registries and long-term clinical follow-up. Many participants have likely accumulated substantial information on healthcare utilisation, medications, rehabilitation services, disability outcomes, and incident neurological diseases throughout their life course. Integration of such data could strengthen the longitudinal analyses and reduce reliance on participant recall. If this is outside the scope of the current project, this could be stated explicitly.

5. The study includes extensive assessments of microbiome composition, metabolomics, intestinal biopsies, PET imaging, and blood biomarkers. A clearer conceptual framework linking intestinal permeability, microbial metabolites, immune activation, and chronic neuroinflammation to long-term recovery and neurodegeneration would help readers understand how these components fit within the overall hypotheses.

6. The current protocol includes multiple primary outcomes spanning neuroinflammation, functional recovery, PET findings, and microbiome composition. A clearer distinction between primary, secondary, and exploratory outcomes would improve interpretability and address concerns regarding multiplicity.

7. The protocol mentions machine-learning approaches for identifying predictors of outcome and disease progression. Given the relatively modest sample size and highly multimodal datasets, further information regarding feature selection, dimensionality reduction, validation procedures, and prevention of overfitting would strengthen the analytical framework.

8. The study involves multiple international collaborators and highly valuable biological samples. It would be useful to clarify whether biological materials will be transferred internationally, whether Material Transfer Agreements (MTAs) and Data Transfer Agreements (DTAs) are in place, and how microbiome and other omics data will be governed under applicable Finnish and European regulations.

Minor Comments:

1. The manuscript should explicitly state whether OVERCOME-TBI should be considered a single-centre study with international analytical collaborators or a genuine multi-centre study, as participant recruitment appears to be centred primarily at Turku University Hospital.

2. The anticipated uptake for colonoscopy and other invasive procedures may require additional justification, particularly given the long follow-up period and the burden of participation.

3. The statistical plan could further clarify how important microbiome confounders, including diet, supplements, smoking, alcohol use, recent travel, and medication exposure, will be incorporated into the analyses.

4. The analytical role of IBS symptoms should be specified, particularly whether IBS will be treated as a confounder, subgroup variable, effect modifier, or outcome of interest.

5. It would be helpful to clarify whether stool microbiome analyses and intestinal wall microbiome analyses will be conducted separately or integrated within a unified analytical framework.

6. The multidisciplinary team is impressive and covers most major domains. However, additional clarification regarding expertise and analytical strategies for machine-learning and multimodal data integration would further strengthen confidence in the proposed analyses.

7. The data-sharing statement could provide more details regarding governance mechanisms, access procedures, and long-term stewardship of the multimodal datasets generated by this project.

8. Although patient and public involvement was not incorporated into the study design, a brief discussion on this decision and potential involvement in dissemination activities would further strengthen the protocol.

VERSION 1 - AUTHOR RESPONSE

Reviewer #1: bmjopen-2026-120552 - "Long-term recovery and microbiota-gut-brain axis disruption after traumatic brain injury (OVERCOME-TBI) – Protocol for a prospective, observational clinical study"

Major Comments:

1. Although OVERCOME-TBI is described as a prospective observational study, it appears to represent a long-term follow-up study based on two previously established prospective cohorts (TBIcare and PACoS-TBI), with additional cross-sectional assessments performed at chronic time points. It would be helpful to explicitly state the study design to facilitate readers' understanding of the analytical framework and interpretation of the findings.
   We thank the reviewer for this observation, which prompted us to state the study design more explicitly. We fully agree that OVERCOME-TBI builds on two previously established prospective cohorts (TBIcare and PACoS-TBI) and adds assessments at chronic time points. After careful consideration and discussion among the co-authors, however, we have chosen to retain the term "prospective" in the title. The great majority of the OVERCOME-TBI data – the comprehensive chronic-phase clinical, neuropsychological, imaging, blood-biomarker, microbiome, and colonoscopy assessments – are newly and prospectively collected under the present protocol, and we believe "prospective observational" most accurately conveys this to readers. We also prefer to avoid the less familiar term "ambispective" in the title itself, to keep it accessible to a broad readership.

   To address the reviewer's underlying point, we have instead clarified the design explicitly in the body of the manuscript. OVERCOME-TBI is now described as a prospective observational study that follows up two previously established prospective cohorts, integrating (i) demographic, clinical, biochemical, imaging, and outcome data already collected during the acute and subacute phases of the original cohorts, which

<u>enable longitudinal comparison, with (ii) new, prospectively collected assessments performed cross-sectionally at chronic time points (2–5 and 12–15 years post-injury). This explicit description has been added to the abstract (Methods and analysis), the first key-message bullet, and the Research materials and methods (Study design and source cohorts) section, so that the longitudinal and cross-sectional elements — and their implications for the analytical framework and interpretation — are clear to readers.</u> We hope the reviewer agrees that this explicit description in the text provides the clarity they rightly sought, while keeping the title concise and widely accessible.

2. Given that the present study integrates two cohorts recruited during different periods, a summary table showing variables collected in each phase would improve transparency. This could include demographics, injury characteristics, functional outcomes, neuropsychological assessments, imaging modalities, biomarkers, gastrointestinal variables, microbiome analyses, and follow-up schedules. Such a table would help readers understand which variables are longitudinally comparable and which assessments are newly introduced.
We agree that a variable-level presentation improves transparency, particularly given the two recruitment periods. We have added Table 1, which lists all assessments by domain (demographics, injury characteristics, functional outcomes, neuropsychological testing, imaging modalities, blood biomarkers and omics, gastrointestinal variables, microbiome analyses, and intestinal tissue sampling) and by study phase (TBIcare acute–subacute, PACoS-TBI acute–subacute, and the OVERCOME-TBI chronic visit), indicating which variables are longitudinally comparable and which are newly introduced at the chronic time point.

   <u>A reference to this table has been added to the Research materials and methods (Study design and source cohorts) section.</u>

3. The protocol would benefit from explicitly stating whether the original studies permitted future re-contact, long-term follow-up, and secondary analyses. Clarification on whether new informed consent will be obtained for all additional procedures, including PET imaging, colonoscopy, microbiome sequencing, and international collaborative analyses, would also be helpful.
We thank the reviewer for raising this important point and have clarified the consent framework. The informed consent obtained in the original TBIcare and PACoS-TBI studies applied to those studies only and did not extend to participation in OVERCOME-TBI. Accordingly, eligible surviving participants are re-contacted by letter and provide new, study-specific written informed consent before any OVERCOME-TBI procedure; this re-contact and re-consent procedure was approved by the regional ethics committee (Wellbeing Services County of Southwest Finland; VARHA/20776/13.02.02/2024). The new consent covers all additional procedures—clinical and neuropsychological assessment, blood sampling and blood-based biomarker and omics analyses, advanced MRI, [^{11}C]PK11195 TSPO-PET imaging, stool collection and faecal microbiome sequencing, and colonoscopy with intestinal biopsies—as well as pseudonymised storage of samples and data and sharing of de-identified data with national and international collaborators under the relevant agreements. Consent is modular, so participants may decline specific procedures (e.g. PET imaging or colonoscopy) while remaining in the study.
<u>The above is now described in the Data handling and record keeping (Subject information and informed consent) section. Governance of international sample and data transfer (MTAs/DTAs) is addressed in our response to Comment 8.</u>

4. Finland has a major advantage in terms of national healthcare registries and long-term clinical follow-up. Many participants have likely accumulated substantial information on healthcare utilisation, medications, rehabilitation services, disability outcomes, and incident neurological diseases throughout their life course. Integration of such data could strengthen the longitudinal

analyses and reduce reliance on participant recall. If this is outside the scope of the current project, this could be stated explicitly.

In OVERCOME-TBI, each participant's history of medical conditions, medications, and injuries is collected by structured interview and then verified and, where necessary, expanded using the electronic health records of the treating hospital system (Wellbeing Services County of Southwest Finland / Turku University Hospital), with the participant's consent, across the departments of neurosurgery, neurology, gastroenterology, and emergency medicine. This reduces reliance on participant recall, and we have added a sentence describing it in the Research materials and methods (Clinical data collection and objectives) section. Linkage to broader national registries is not part of the current consented protocol, as it would require separate register-holder permissions.

<u>The use of electronic medical records has now been clarified in the manuscript.</u>

5. The study includes extensive assessments of microbiome composition, metabolomics, intestinal biopsies, PET imaging, and blood biomarkers. A clearer conceptual framework linking intestinal permeability, microbial metabolites, immune activation, and chronic neuroinflammation to long-term recovery and neurodegeneration would help readers understand how these components fit within the overall hypotheses.

<u>We have added a conceptual framework to the Introduction (referring to Figure 1) that describes the proposed pathway. We also map each study component to this pathway, clarifying how the assessments relate to the study's two hypotheses.</u>

6. The current protocol includes multiple primary outcomes spanning neuroinflammation, functional recovery, PET findings, and microbiome composition. A clearer distinction between primary, secondary, and exploratory outcomes would improve interpretability and address concerns regarding multiplicity.

This is an important remark, thank you. The primary and secondary outcomes were prespecified and registered on ClinicalTrials.gov (NCT06927063) and in the ethics application. The four primary outcome domains are treated as co-primary, each addressing a component of the two study hypotheses rather than a single principal endpoint; the advanced imaging measures are secondary.

<u>To improve interpretability, we have clarified this structure and designated the integrative cross-modal and machine-learning analyses as exploratory and hypothesis-generating. Multiplicity across the high-dimensional, multimodal outcomes is controlled by false discovery rate correction, as described in the Statistics section.</u>

7. The protocol mentions machine-learning approaches for identifying predictors of outcome and disease progression. Given the relatively modest sample size and highly multimodal datasets, further information regarding feature selection, dimensionality reduction, validation procedures, and prevention of overfitting would strengthen the analytical framework.

As the appropriate methods depend on the specific data and question, the detailed feature selection, dimensionality reduction, and validation procedures will be pre-specified for each substudy in collaboration with statisticians and systems biologists. However, it is important to describe the multimodal data integration in the protocol.

<u>We have added a short "Multimodal data integration and machine learning" subsection to the statistical plan. It states that, given the relatively modest sample size and high dimensionality, machine-learning analyses are exploratory and hypothesis-generating, and that overfitting is limited by reducing dimensionality before modelling, keeping models as simple as the data allow, and evaluating performance by cross-validation, with confirmation sought in the second cohort or external data (e.g. via the InTBIR consortium) where possible.</u>

8. The study involves multiple international collaborators and highly valuable biological samples. It would be useful to clarify whether biological materials will be transferred internationally, whether Material Transfer Agreements (MTAs) and Data Transfer Agreements (DTAs) are in

place, and how microbiome and other omics data will be governed under applicable Finnish and European regulations.

The study analyses will be performed by collaborating laboratories in Finland and abroad, and the specific laboratories may change over the course of the study as analytical platforms and collaborations evolve. In every case, transfer of biological material is governed by a Material Transfer Agreement and transfer of personal data by a Data Transfer/Processing Agreement, established before any sample or dataset leaves the study centre: no such contracts have yet been executed.

<u>We have added a paragraph to the "Data management" section clarifying international transfer and governance.</u>

<u>Minor Comments:</u>

9. The manuscript should explicitly state whether OVERCOME-TBI should be considered a single-centre study with international analytical collaborators or a genuine multi-centre study, as participant recruitment appears to be centred primarily at Turku University Hospital.

OVERCOME-TBI is a single-centre study with international analytical collaboration: all participant recruitment, clinical assessment, imaging, and sample collection are conducted at Turku University Hospital, while biomarker, omics, and other specialised analyses are carried out by national and international collaborating laboratories.

<u>We have now stated this in the "Study design and source cohorts" section.</u>

10. The anticipated uptake for colonoscopy and other invasive procedures may require additional justification, particularly given the long follow-up period and the burden of participation.

The participants are drawn from well-characterised cohorts with an established relationship with Turku University Hospital, which supports engagement over long follow-up. The risks, burden, and potential benefits of colonoscopy and biopsies – including detection and immediate treatment of incidental findings such as advanced polyps – are described transparently in the patient information sheet that participants read before giving consent, and this was reviewed and approved by the regional ethics committee.

<u>We have added a justification of the anticipated uptake and also note that our recruitment and uptake estimates were set conservatively.</u>

11. The statistical plan could further clarify how important microbiome confounders, including diet, supplements, smoking, alcohol use, recent travel, and medication exposure, will be incorporated into the analyses.

These confounders are recorded at the study visit (diet, dietary supplements, smoking, alcohol use, recent travel, and recent medication, including antibiotics, please see Research materials and methods (Clinical data collection and objectives).

<u>We have now also clarified in the statistical plan that the microbiome models are adjusted for these variables, and that recent antibiotic or probiotic use is additionally addressed through the pre-specified sensitivity analyses.</u>

12. The analytical role of IBS symptoms should be specified, particularly whether IBS will be treated as a confounder, subgroup variable, effect modifier, or outcome of interest.

In OVERCOME-TBI, Rome IV-defined IBS symptoms are treated as a phenotype/outcome of interest (comparing patients with TBI and orthopaedic controls and relating IBS symptoms to the microbiome and calprotectin) and as a stratifying subgroup variable (IBS-symptomatic versus asymptomatic) for the microbiome, histology, and metabolomic comparisons. In the gut–brain analyses, IBS symptoms are additionally explored as a potential effect modifier. They are not treated as a confounder to be adjusted away.

<u>We have now stated this in the Statistical plan and analysis section.</u>

13. It would be helpful to clarify whether stool microbiome analyses and intestinal wall microbiome analyses will be conducted separately or integrated within a unified analytical framework.
Both stool (faecal) and intestinal wall microbiome samples are processed and sequenced at the Department of Clinical Microbiology, Turku University Hospital. DNA extraction will be performed using optimal methods for each sample material. Sequencing and bioinformatics pipelines will be harmonised to ensure comparability for all sample material. The two compartments are analysed as separate datasets and then compared within this common framework, because the microbes in stool and those attached to the intestinal wall differ and provide complementary information.
We have clarified the above in the Statistical plan and analysis (Faecal microbiome analyses) section.
14. The multidisciplinary team is impressive and covers most major domains. However, additional clarification regarding expertise and analytical strategies for machine-learning and multimodal data integration would further strengthen confidence in the proposed analyses.
We thank the reviewer for this remark. The current coauthors are the researchers essential to the study's design and conduct. Because the design is complete and recruitment has only just begun, the specific individuals who will lead every analytical step have not all yet been invited and are not yet fixed. We would emphasise, however, that the multidisciplinary coauthor list comprises investigators with extensive national and international collaborative networks, which ensures that the appropriate statistical, machine-learning, and data-integration expertise will be engaged as each analysis is undertaken.
We have added a sentence to the Multimodal data integration and machine learning subsection under the Statistical plan and analysis part to reflect this, and, as noted in our response to Comment 7, the detailed methodology for each substudy will be pre-specified together with the relevant experts.
15. The data-sharing statement could provide more details regarding governance mechanisms, access procedures, and long-term stewardship of the multimodal datasets generated by this project.
Requests will be assessed by the study steering group and, if approved, fulfilled under data-haring and material-transfer agreements, consistent with participant consent and applicable Finnish and EU data-protection legislation. The data are held on the institutional servers of the University of Turku and Turku University Hospital, which provide long-term stewardship, and the scope and format of any shared dataset will depend on the individual request.
We support open science and have expanded the data-sharing statement accordingly, while noting that the details necessarily depend on the specific requests received.
16. Although patient and public involvement was not incorporated into the study design, a brief discussion on this decision and potential involvement in dissemination activities would further strengthen the protocol.
We thank the reviewer. Patient and public involvement was not incorporated into the study design because OVERCOME-TBI is a long-term follow-up of two previously recruited, extensively phenotyped cohorts, with a complex multimodal assessment protocol designed by the research team.
We have now clarified that patients and the public will be engaged in dissemination through accessible channels such as public lectures, social media, podcasts and newspaper articles, aimed at raising awareness of the consequences and prevention of TBI.

VERSION 2 - REVIEW

**Reviewer**	**1**
**Name**	**Pongpirul, Krit**
**Affiliation**	**Chulalongkorn University Faculty of Medicine, Preventive and Social Medicine**
**Date**	**06-Sep-2026**
**COI**	

Thank you for the comprehensive revision. Most of my previous comments have been adequately addressed.

A few minor points remain:

1. Please carefully clean the manuscript for revision artifacts, including duplicated text and typographical errors such as “aan observational” and “biopsiesand colonoscopy biopsies.”

2. Please use consistent terminology for the study design throughout the manuscript. The revised description is clear, but the Statistics section still uses a less consistent description of the study as partly cross-sectional and partly longitudinal.

3. Table 1 is useful, but the distinction between samples being “biobanked” and measurements actually being performed at the chronic visit could be made clearer.

VERSION 2 - AUTHOR RESPONSE

Reviewer’s Comments to Author: bmjopen-2026-120552 – “Long-term recovery and microbiota–gut–brain axis disruption after traumatic brain injury (OVERCOME-TBI) – Protocol for a prospective, observational clinical study”

Thank you for the comprehensive revision. Most of my previous comments have been adequately addressed. A few minor points remain:

We thank the reviewer for the positive assessment. We have addressed the three remaining points as detailed below and have carefully re-read the entire manuscript.

Please carefully clean the manuscript for revision artifacts, including duplicated text and typographical errors such as “aan observational” and “biopsiesand colonoscopy biopsies.”

We have carefully proofread the entire manuscript to remove revision artefacts, duplicated text, and typographical errors. In particular, we corrected “aan observational” to “an observational” and the “biopsiesand colonoscopy biopsies” text to “and colonoscopy biopsies”, completed a sentence that had been truncated during a previous revision, and removed a duplicated summary sentence in the Article Summary. All tracked changes have now been accepted so that no markup artefacts remain in the submitted version.

Please use consistent terminology for the study design throughout the manuscript. The revised description is clear, but the Statistics section still uses a less consistent description of the study as partly cross-sectional and partly longitudinal.

We have harmonised the study design terminology throughout the manuscript. The Statistics (Sample size – overall study) section previously described the study as “observational cross-sectional and, on the other hand, partially longitudinal”; it now

uses exactly the same wording as the abstract and the Research materials and methods (Study design and source cohorts) section, namely "a prospective, observational study with longitudinal follow-up and a cross-sectional chronic-phase component".

Table 1 is useful, but the distinction between samples being "biobanked" and measurements actually being performed at the chronic visit could be made clearer.

We have revised Table 1 accordingly. First, we added a legend defining each status term and, in particular, distinguishing "Biobanked" (a biological sample collected and stored at the visit, with the assay performed later) from an assessment actually performed at that phase ("Yes"). Second, for the blood protein biomarkers and blood omics, the OVERCOME-TBI (chronic) cells now read "Sample collected at chronic visit, biobanked", making it explicit that the sample is stored and analysed subsequently rather than measured at the chronic visit.
