# Supplementary material for: Long-term recovery and microbiota–gut–brain axis disruption after traumatic brain injury (OVERCOME-TBI): protocol for a prospective, observational clinical study
Source: BMJ Open. 2026 Sep 23;16(9):e120552. doi: 10.1136/bmjopen-2026-120552 (PMC13630022; doi:10.1136/bmjopen-2026-120552)
Supplement: Supplementary file 2 [file bmjopen-16-9-s001.docx]

**Appendix.** Full MRI protocol summary table for OVERCOME-TBI

| **Sequence** | **Mode** | **Voxel/Slice (mm)** | **Matrix** | **TR (ms)** | **TE (ms)** | **Key Parameters** | **Purpose** |
| --- | --- | --- | --- | --- | --- | --- | --- |
| 3D T1 MPRAGE | 3D Sag | 0.5 iso | 256×256 | - | - | TI 900, FA 8°, ZIP512 | Structural, brain age |
| Ax T2 FSE | 2D Ax | 4.0 + 0.4 gap | 384×320 | 7200 | 89 | ETL 18, 36 slices | Structural |
| 3D T2 FLAIR | 3D Sag | 0.5 iso | 256×256 | 6000 | 105 | TI 1768, ETL 200, HS 1.4 | WM lesions |
| DTI b=1000 | 2D Ax | 2.0 iso | 128×128 | 6200 | 72 | 9 dir, 3 b0, HB×2 | DTI metrics |
| DTI b=1500 | 2D Ax | 2.0 iso | 128×128 | 6200 | 72 | 30 dir, 3 b0, HB×2 | NODDI, CSD |
| DTI b=2800 | 2D Ax | 2.0 iso | 128×128 | 6200 | 72 | 64 dir, 3 b0, HB×2 | NODDI, CSD |
| 3D SWI | 3D Ax | 1.0 iso | 288×256 | Min | 25 | 7 echoes, FA 15° | Microhaemorrhages |
| rs-fMRI | 2D Ax | 3.4 iso | 64×64 | 920 | 30 | 900 vol, HB×4, FA 70° | Functional connectivity |

Ax, axial; b, diffusion weighting (b-value, s/mm²); b0, non–diffusion-weighted image (b = 0 s/mm²); CSD, constrained spherical deconvolution; dir, diffusion-encoding directions; DTI, diffusion tensor imaging; ETL, echo train length; FA, flip angle; FLAIR, fluid-attenuated inversion recovery; FSE, fast spin echo; HB, HyperBand (simultaneous multi-slice acceleration factor); HS, HyperSense (acceleration factor); iso, isotropic; Min, minimum; MPRAGE, magnetisation-prepared rapid gradient echo; NODDI, neurite orientation dispersion and density imaging; rs-fMRI, resting-state functional magnetic resonance imaging; Sag, sagittal; SWI, susceptibility-weighted angiography; T1, T1-weighted; T2, T2-weighted; TE, echo time; TI, inversion time; TR, repetition time; vol, volumes; WM, white matter; ZIP512, zero-fill interpolation to a 512 × 512 matrix.
